# Water Added Probiotics Attenuates Sumithion‐Induced Toxicity on Growth, Intestinal Deformities, Erythrocytic Abnormalities, and Immunity in Nile Tilapia (*Oreochromis niloticus*)

**DOI:** 10.1155/anu/2689798

**Published:** 2026-01-16

**Authors:** Atika Anjum, Taslima Parvin, Shaila Sultana, Azmaien Naziat, Zannatul Ferdous, Md. Mahiuddin Zahangir, Md Shahjahan

**Affiliations:** ^1^ Department of Fish Biology and Biotechnology, Faculty of Fisheries, Chattogram Veterinary and Animal Sciences University, Chattogram, 4225, Bangladesh, cvasu.ac.bd; ^2^ Department of Fisheries Management, Faculty of Fisheries, Bangladesh Agricultural University, Mymensingh, 2202, Bangladesh, bau.edu.bd

**Keywords:** antioxidant, growth performance, immunity, Nile tilapia, oxidative stress, probiotics, sumithion

## Abstract

Probiotics are important microflora that help in improving gut health, enhancing immunity, and boosting overall well‐being. Sumithion (O, O‐Dimethyl O‐(3‐methyl 4‐nitrophenyl) phosphorothioate) is an organophosphate insecticide widely used in agriculture and aquaculture, has harmful effects in aquatic organisms due to its indiscriminate use. Therefore, this study evaluates the counteracting effects of multispecies probiotics (*Bacillus subtilis*, *B. thuringiensis Lactobacillus plantarum*, and *L. buchneri*) to sumithion toxicity in Nile tilapia (*Oreochromis niloticus*). Juvenile (12.84 ± 0.09 g) Nile tilapia were reared with four treatment groups: control, sumithion (0.3 mg/L), probiotics (1.0 mL/L), and sumithion + probiotics (0.3 mg/L + 1.0 mL/L) with three replicates for 42 days. Results showed that fish exposed to sumithion had significantly lowered weight gain (WG) and specific growth rate (SGR), while probiotics incorporation improved the growth performance. Probiotics mitigated sumithion‐induced effects by increasing hemoglobin (Hb) and decreasing glucose (Glu) levels, while also reducing the higher erythrocytic abnormalities. Sumithion exposure caused marked alterations in intestinal morphology, and these changes were partially restored by co‐administrations of multispecies probiotics, while enhanced intestinal mucosal folds, improving goblet and enterocytes cell numbers, and widen the lamina propria. Significantly higher and lower levels of mRNA for growth hormone (GH; *gh*) and insulin‐like growth factors (IGFs; *igf-1* and *igf-2*) genes were found in probiotics and sumithion‐exposed fish, respectively. Relative mRNA level for antioxidant genes (catalase [*CAT*] and superoxide dismutase [*SOD*]) was significantly increased in fish exposed to sumithion, while the nonsignificant differences was observed in probiotics and sumithion and probiotics treated fish. Conversely, the expression of immune‐related genes (tumor necrosis factor alpha [*TNF-α*], interleukin beta [*IL-1β*], and interferon gamma [*IFN-γ*]), was downregulated in sumithion‐treated fish, and relative mRNA levels increased following the addition of probiotics. Therefore, incorporating probiotics into the aquatic environment demonstrated beneficial effects on haemato‐biochemical properties, erythrocyte structure, and immune function, ultimately enhancing growth and countering the stress induced by sumithion pesticides.

## 1. Introduction

Sumithion (O, O‐Dimethyl O‐(3‐methyl 4‐nitrophenyl) phosphorothioate) is organophosphates insecticides in aquaculture inhibiting acetylcholinesterase and disrupting neural function in insects and aquatic organisms [1]. The indiscriminate use of sumithion in agriculture and aquaculture leads to their runoff into aquatic systems, where they accumulate and adversely affects aquatic plants and organisms, particularly fish species [2]. These accumulation disrupts fish immune systems, causes immunotoxicity, alters ecosystem balance, and reduced fish populations [3]. The lipophilic nature of sumithion allows accumulation of fish internal organs (liver), impairing growth by disrupting metabolism, and also alters behavioral signs, including abnormal locomotion and rapid gill movement [4, 5]. Moreover, sumithion has sublethal effects on fish, such as developmental abnormalities in zebrafish, *Danio rerio* [5], and hematobiochemical disturbance in striped catfish (*Pangasianodon hypophthalmus*) [6]. Hence, further research is essential to explore the strategies for reducing the harmful impact of sumithion on the growth, immunity, and antioxidant system of fish.

Goblet cells (GCs) in the intestine secretes mucin glycoproteins that form the protective mucus layer, a key component of the hosts innate and adaptive defenses depends on the synchronizing regulatory pattern of the intercellular junctions, epithelial cells, and immune response [7, 8]. Previous studies found that supplementation of *Lactobacillus* spp. was reported to stimulate mucin secretion and prevent the adherence of enteropathogenic bacteria, increasing the mucus layer as a protection from pathogen invasions [9]. In contrast, pesticide exposure affects the gastrointestinal tract by degenerating brush borders, hyperplasia of GCs, and enlargement and disintegration of lamina propria in adult zebra fish [10]. While, probiotics feed additive improves intestinal structures and lymphocyte number, and enhancing digestive activity of *Onchorchynus mykiss* [11]. Thus, studying the GCs and associated intestinal immune structures in the gastrointestinal tract will, therefore, essential to understand the counteract effects of probiotics against the toxic substances in fish.

Growth hormone (GH), a monomeric polypeptide synthesized by the anterior pituitary gland, regulates growth, development, and physiological activities [12]. Additionally, insulin‐like growth factors (IGFS; IGF‐I and IGF‐II) modulates GH secretion and promote cell proliferation, growth, and survival through signaling pathway mediated by the IGF receptors within the hypothalamic–pituitary–peripheral (HPP) axis [13, 14]. Sumithion can act as an endocrine‐disrupting chemical (EDC) that ultimately interferes with the HPP axis by reducing the secretion of GH and IGF‐1 which ultimately disrupts the growth process of fish. However, the disruption caused by sumithion can be upregulated the expression of GH and IGF‐1 in fish by incorporating probiotics [15]. In addition, probiotics administration is beneficial in increasing immunity as well as growth by stimulating GH synthesis in the fish [16]. Hence, it is essential to evaluate the GH–IGF system in fishes having exposure to pesticides to understand the adverse impacts and find out possible ways to alleviate them.

Cytokines are a type of polypeptide of small molecular structure that acts as a signaling molecule involved in immune system activation, leading to inflammation stimulation to minimize stress or manage pathogen levels in fish [17]. Studies suggested that interleukin beta (IL‐1*β*) acts as the initial immune barrier under normal circumstances, but is disrupted by stressors, and there can be changes in the expression [18]. In addition, interferons (IFNs), a group of cytokine molecules, have a significant impact in establishing an antiviral state within cells in response to viral infections, facilitating tumor surveillance, activating defense mechanisms, and modulating the activity and function of immune cells [19]. Furthermore, tumor necrosis factor alpha (TNF‐*α*) is crucial for several physiological functions, such as inflammation, immune responses, and cell death. Thus, cytokines are considered beneficial indicators of immune responses in teleosts under various adverse conditions like pesticide or any other chemical exposure [20]. In *Cyprinus carpio*, organophosphates like azadirachtin (AZA) resulting in upregulated expression of cytokines like *TNF-α* and *IL-1β* [21]. Research shows that the application of probiotic can modify the expression of inflammatory mediators like *IL* and *TNF*‐*α* [22, 23]. However, there is a gap in the understanding of how probiotics counteracts the immunological and cytokine responses induced by pesticides exposure in fish. Hence, a comprehensive knowledge of the collective influences of probiotics and pesticides on the ontogeny of immune responses and cytokine profiles is crucial to overcome the acute repercussions of pesticide proximity in aquatic ecosystems.

Oxidation, through which free radicals and hydrogen peroxides are synthesized, is suppressed by antioxidants. The produced free radicals and hydrogen peroxides result in oxidative damage under stress conditions, which are detoxified by the antioxidant enzymes such as superoxide dismutase (SOD), catalase (CAT) and glutathione peroxidase (GPx) [24]. Reactive oxygen species (ROS) are activated with contact to pesticides, leading to stimulation of oxidative stress, inflammation, immunotoxicity, and genotoxicity [25]. However, prior research has shown that the expression levels of *TNF-α*, *IL-1β*, *IFN-γ*, *SOD*, and *CAT* vary in response to a variety of probiotic supplement under various stress [26, 27]. Thus, probiotics might also have a mitigating impact on pesticide exposure on fish.

Probiotics are the beneficial living microorganisms, have become an integral part in aquaculture by improving immunity against disease, mitigating the limitations, and negative impacts of antimicrobial agents [28, 29]. Nevertheless, multispecies probiotics exerted more beneficial synergic effects, but the combined forms of probiotics should complement each other for the desired effects in immune stimulation [30]. For instance, probiotics, such as *Bifidobacterium longum*, *L. acidophilus*, *L. lactis*, and *Lactobacillus paracascei*, were reported to upregulate the cytokine expressions in fish [31], and various groups of *Bacillus sp*. tends to elevate inflammatory reaction in the intestine [32]. Despite these beneficial functions in aquaculture, there is a lack of knowledge on choosing suitable beneficial bacteria strains for specific fish and shellfish species, especially in reducing the toxic effect of pesticides. Thus, further exploration of probiotic potential is essential to identify a highly effective and sustainable approach for mitigating the toxic effect of sumithion and enhancing fish gut health, boosting overall well‐being, and a sustainable aquaculture system.

Nile tilapia (*Oreochromis niloticus*) is an euryhaline fish belonging to the family Cichlidae, found in a divergent habitat of freshwater and brackish water [33]. Nile tilapia is a suitable candidate for aquaculture for its adaptability to high stocking density, low production cost, ability to adapt to different tropical water levels, and high disease resistance [34]. Nile tilapia is the second most widely farmed freshwater fish globally exceeding 6 MT, driving a rapidly expanding aquaculture industry to meet both domestic and international demand [35, 36]. However, limited research exists on the mitigating strategies of sumithion exposure in *O. niloticus*. Therefore, this research focused to investigate the protective effects of multispecies probiotics against sumithion‐induced toxicity in *O. niloticus*, focusing on growth performance, immune responses, antioxidant defense, gut histology, and cytokine expression. Thus, simultaneously evaluating multiple physiological, immunological, and intestinal parameters to uncover the mechanisms by which probiotics mitigate pesticide‐induced stress, addressing a critical knowledge gap in sustainable aquaculture practices.

## 2. Materials and Methods

### 2.1. Experimental Fish

Juvenile monosex *O. niloticus* (9.0 ± 0.5 cm in length and weighing 12.84 ± 0.09 g) were initially acquired from Bangladesh Fisheries Research Institute (BFRI), Mymensingh, Bangladesh, and brought to the wet laboratory of fish ecophysiology, Department of Fisheries Management, Bangladesh Agricultural University, Bangladesh with aeration. Selected fish were ensured of their physical fitness and free from disease by physical examination. Acclimatization of collected *O. niloticus* was performed over 10 days in a large concrete tank (500 L) provided with dechlorinated water and a continuous air supply. Fish were supplied with commercial pellet feed (Mega Fish Feed Ltd., Bangladesh), containing 32% crude protein, twice a day until the fish were fully satiated.

### 2.2. Experimental Design

*O. niloticus* (*n* = 20) was stocked in 12 glass aquaria (each with a volume of 150 L and dimensions of 75 × 45 × 45 cm^3^, containing 100 L of freshwater. After acclimatization, fish were exposed to four different treatments includes; T1 (control, no sumithion or probiotics), T2 (sumithion, 0.3 mg/L), T3 (probiotics, 1.0 mL/L), and T4 (sumithion, 0.3 mg/L + probiotic, 1 mL/L) with three replicates set up for each treatment for 42 days under the maintenance of natural photoperiod (14:10, light: dark). Multispecies probiotics developed in the laboratory of fish ecophysiology, BAU, Mymensingh, containing *Bacillus subtilis* (5 × 10^9^ colony‐forming unit [cfu]/mL), *B. thuringiensis* (4 × 10^9^ cfu/mL), *Lactobacillus plantarum* (5.8 × 10^9^ cfu/mL), and *L. buchneri* (6.5 × 10^9^ cfu/mL), selected based on their role in improving growth and immunity [37], were incorporated into water at the rate of 1.0 mL/L. The probiotics dosage was selected based on the previous studies, where probiotics incorporation successfully improved growth, gut morphology, and stress tolerance [38]. The reared fish were provided with commercial floating pellet feed (Mega Fish Feed Ltd., Bangladesh), containing 32% crude protein, 31% carbohydrate, 6.2% fat, 5.4% fiber, 10.4% ash, and 2.9% mineral per day the equivalent to 5% of their body weight (BW) at 9:00 am and 5:00 pm until the appearance of full satiation. With increased in body size, fish were fed 3% of their BW with the feed ration adjusted accordingly. Water quality parameters, including temperature (°C), ammonia concentration (mg/L), and pH were regularly monitored to ensure they remained within the optimum ranges. No fish mortalities were found in any treatment group during the study period. All experimental procedures were carried out following the guidelines established by the Animal and Ethical committee of Bangladesh Agricultural University, Mymensingh, Bangladesh (Approval No. Bau‐FoF/2002/003).

### 2.3. Sampling and Data Collection

After being treated with different concentration of sumithion and probiotics, *O. niloticus* was sacrificed at the end of rearing periods after being anesthetized with 5 mg/L clove oil, and their total length (TL) and BW for all the collected individuals were recorded [38]. Afterwards, the fish were sacrificed by pithing method at the head and the liver and pituitary glands were collected (*n* = 6) randomly. Collected liver and pituitary were immediately preserved in RNAlater (Ambion, Austin, TX) and stored at 4°C for 24 h. The samples were then preserved at −80°C for further RNA extraction. Intestinal samples were collected by dissecting the abdominal cavity, afterwards the intestinal tract was separated, weighed, and fixed in Bouin’s fixative, followed by storage in 70% ethanol after 24 h for histological analysis of intestinal structure [37].

### 2.4. Growth Performance of Nile Tilapia

The growth performance, including weight gain (WG; g), percent WG (%), specific growth rate (SGR, %/day), feed conversion ratio (FCR), and survival (%), were calculated by using the following equation mentioned in the previous studies [27]:

Weight gain (WG)=Final body weight (FBW) (g) − initial body weight (g),

Percent weight gain= Final body weight (g) −initial body weight(g)Initial body weight (g)×100,

Specific growth rate− SGR %/day= Ln final weight g−ln  initial weight g Number of days ×100,

FCR=Feed given (dry weight in g)Body weight gain (wet weight in g),

Survival %=Final number of harvested fishInitial number of fish×100.

### 2.5. Hematobiochemical Analysis

After exposure to different treatments, fish blood samples (*n* = 6) were collected from the caudal vein into an anti‐coagulant tube. Afterwards, the hemoglobin (Hb; g/dL) and glucose (Glu; mg/dL) levels were measured by using a digital EasyTouch GCHb, Blood Glu/Hb dual‐function monitoring system (Model: ET‐232, Bioptik Technology Inc., Taiwan 3505–7).

Erythrocytic cellular abnormalities (ECAs) and erythrocytic nuclear abnormalities (ENAs) were identified, denoted, and the frequency distribution of these abnormalities were analyzed according to the protocol described previously [5]. Briefly, from collected blood samples (*n* = 6), two blood smears for each sample were prepared following several steps; 5 µL of collected blood was immediately smeared on a glass slide and air dried for 30 min. After air drying, smears were fixed with 100% ethanol for 10 min and stained with 5% Giemsa stain solution for another 10 min. After rinsing with distilled water, slides were air dried and the frequency of abnormalities was observed under a microscope (Carl ZEISS Microscope, Gmblt, OpticaB‐190 series, USA).

### 2.6. Histopathological Observation of the Intestine

For the histopathological observation, paraffin‐embedded hematoxylin–eosin staining based procedure was followed [38]. Samples fixed by Buoin’s fixative was dehydrated by using a series of graded alcohol (70%, 80%, 90%, 95%, and 100%) solutions, each for 2−3 h, consecutively, followed by cleaning with xylene (100%). The samples were kept overnight, then infiltrated with paraffin wax for embedding, followed by placing them in a hot air oven overnight for proper embedding of paraffin wax into the sample. The next day, blocks were prepared where samples were placed and allowed to air dried for 24 h. The block was sectioned using a microtome machine (KD2258, China). Those sections were air‐dried, and stained the slides using hematoxylin and eosin. After air drying, the stained slides were applied with 1 drop of Dibutyl Phthalate Polystyrene Xylene (DPX) (Qualikems Fine Chemical PVT Ltd., India) for mounting the prepared slides. A thin cover slip was placed on the mounted slides, which were allowed to set, and subsequently analyzed under a microscope (Carl ZEISS Microscope, Gmblt, OpticaB‐190 series, USA).

### 2.7. RNA Extraction, cDNA Preparation, and Real‐Time PCR Assays

Total RNA was extracted from the liver and pituitary sample using TRIzol Reagent (Invitrogen, Thermo Fisher, USA) in accordance with the manufacturer’s instructions [26]. The RNA pellets were subsequently dried and 20 µL of DEPC (Diethyl polycarbonate) water was added to dissolve the RNA pellet and stored at −80°C for further use after incubating at 55°C. Nanodrop spectrophotometer (Nanophotometer NP80, Germany) was used to evaluate the RNA integrity and quality by a ratio of 260/280 and agarose gel electrophoresis. The measured value of approximately 2.0 was used for cDNA synthesis. Then the first strand of cDNA was synthesized using 500−1000 ng total RNA with a cDNA synthesis kit (AddScript, Korea) as per the manufacturer’s instructions. To synthesize cDNA, a 20 µL mixture containing RNA template, 4 µL of 5x buffer, 2 µL of dNTP mixture, 2 µL of 10x oligo dT primer, 1 µL of enzyme, and nuclease‐free distilled water was prepared and incubated following a temperature cycle (10 min at 25°C for priming, 60 min at 50°C for RT reaction, and 80°C for 5 min for enzyme deactivation) in a water bath and preserved at −20°C for subsequent use.

Real‐time PCR was carried out with the QuantStudio 5 Real‐time PCR system (Model: 7500, Applied Biosystem, USA). Primers for measuring *gh*, *igf-1*, *igf-2*, *SOD*, *CAT*, *IFN*‐*γ*, *TNF-α*, and *IL-1*‐*β* are listed in Table 1. The reaction mixtures (10 µL) containing standard cDNA or sample (1 µL), forward and reverse primers (0.4 µL each), and SYBR Master (Addbio Inc, Korea) were amplified at 95°C for 30 s, followed by 40 cycles at 95°C for 5 s, 60°C for 30 s, and 72°C for 30 s. Melting curve analysis were performed on the final product to validate the precise amplification of each cDNA. The expression levels were normalized by the 2^−∆∆CT^ method with *β-actin* as the housekeeping gene. All qPCR experiments were conducted in duplicate.

**Table 1:** List of primers used in the real‐time PCR.

Primers		Nucleotide sequences	Temperature (°C)	References
*gh*	F	5′CTGCTGATCAGGGCCAATC3′	58	[39]
	R	5′TCGACATTTAGCTACCGTCAGG3′	—	
*igf-1*	F	5′GTTTGTCTGTGGAGAGCGAGG3′	58	[39]
	R	5′GAAGCAGCACTCGTCCACG3′	—	
*igf-2*	F	5′GCTTTTATTTCAGTAGGCCAACCA3′	58	[39]
	R	5′CACAGCTACAGAAAAGACACTCCTCTA3′	—	
*SOD*	F	5´ GACGTGACAACACAGGTTGC 3´	57	[40]
	R	5´ TACAGCCACCGTAACAGCAG 3´	—	
*CAT*	F	5´ TCAGCACAGAAGACACAGACA 3´	57	[40]
	R	5´ GACCATTCCTCCACTCCAGAT 3´	—	
*TNF-α*	F	5´ GGAAGCAGCTCCACTCTGATGA 3´	59	[40]
	R	5´ CACAGCGTGTCTCCTTCGTTCA 3´	—	
*IL-1β*	F	5′ CAAGGATGACGACAAGCCAACC 3′	55	[41]
	R	5′ AGCGGACAGACATGAGAGTGC 3′	—	
*IFN-γ*	F	5′ TGACCACATCGTTCAGAGCA 3′	55	[41]
	R	5′ GGCGACCTTTAGCCTTTGT 3′	—	
*β-actin*	F	5´ CGAGCTGTCTTCCCATCCA 3´	57	[42]
	R	5´ TCACCAACGTAGCTGCTTTCTG 3´	—	

### 2.8. Statistical Analysis

All the data representing growth performance, intestinal structures and gene expressions are expressed as mean ± standard deviation (SD). Data were first analyzed using one‐way analysis of variance (ANOVA) to assess the significance comparing the three‐treatment groups with the control group. Upon observing any significance, the means were compared using Tukey’s HSD post hoc test in SPSS (Version 30.0). Visualizations were produced using packages “ggplot2” [43] and “ggpubr” [44] in R (R version 4.4.3). Statistically significant differences were indicated by *p* < 0.05, unless otherwise specified in the text.

## 3. Results

### 3.1. Growth Performance of *Oreochromis niloticus*

At the end of the experimental period, WG of fish in T2 (sumithion) were significantly reduced (*p* < 0.05), while fish incorporated with probiotics in both T3 (probiotics) and T4 (sumithion + probiotics) had significantly (*p* < 0.05) higher WG compared to T1 (control) (Table 2). Similarly, %WG and SGR was significantly (*p* < 0.05) higher in fishes reared with probiotics (T3), followed by T4 (sumithion + probiotics) and lowest (*p* < 0.05) in T2 (sumithion) group. On the contrary, FCR showed an opposite trend among these treatments. Significantly higher FCR were observed in T2 (sumithion) group compared to other treatments, having the lowest FCR in T3 (probiotics), followed by T4 (sumithion + probiotics). The survival rate was unaffected in all treatments (Table 2).

**Table 2:** Growth response of Nile tilapia (*O. niloticus*) reared with sumithion, probiotics, and sumithion + probiotics for 42 days (*n* = 12).

Parameters	Treatments			
	Control (T1)	Sumithion (T2)	Probiotics (T3)	Sumithion + probiotics (T4)
IBW (g)	12.84 ± 0.09^a^	12.84 ± 0.09^a^	12.84 ± 0.09^a^	12.84 ± 0.09^a^
FBW (g)	42.39 ± 1.89^c^	36.11 ± 2.07^d^	54.93 ± 3.03^a^	47.28 ± 3.56^b^
WG (g)	29.54 ± 1.89^c^	23.28 ± 2.06^d^	42.78 ± 3.56^a^	34.79 ± 3.36^b^
%WG	229.83 ± 14.67^c^	181. 26 ± 16.08^d^	352.09 ± 24.95^a^	268.11 ± 26.17^b^
SGR (%/day)	1.23 ± 0.05^c^	1.07 ± 0.06^d^	1.56 ± 0.55^a^	1.35 ± 0.07^b^
FCR	2.23 ± 0.14^a^	3.11 ± 0.28^c^	1.53 ± 0.10^b^	1.76 ± 0.16^c^
Survival rate (%)	100	100	100	100

*Note:* All values (*n* = 12) presented as mean ± standard deviation (SD). Different lowercase letters in superscript indicate a significant (*p* < 0.05) difference among different treatments.

Abbreviations: %WG, percent weight gain; FBW, final body weight; FCR, feed conversion ratio; IBW, initial body weight; SGR, specific growth rate; WG, weight gain.

### 3.2. Changes in Hematobiochemical Parameters

In the current experiments, fish exposed to sumithion (T2) demonstrated a significant decrease (*p* < 0.05) in Hb levels in relative to the control group (T1) (Figure 1A). Oppositely, probiotic supplementation accelerated the Hb values in fishes. Fishes supplemented with probiotics (T3) had significantly higher (*p* < 0.05) Hb levels compared to T2 (sumithion), having no statistical difference with control group (T1). Furthermore, fish receiving combined supplementation of sumithion and probiotics (T4) exhibited intermediate Hb levels, which was higher than T2 (*p* < 0.05) and lower than T1 and T3 (Figure 1A).

Figure 1Alteration in (A) hemoglobin (Hb) and (B) glucose (Glu) levels of Nile tilapia (*O. niloticus*) treated with sumithion, probiotics, and sumithion + probiotics for 42 days. Values are presented as mean ± standard deviation of the mean (*n* = 12). Different lowercase letters indicate a significant (*p* < 0.05) difference among various treatments.

**Figure:**
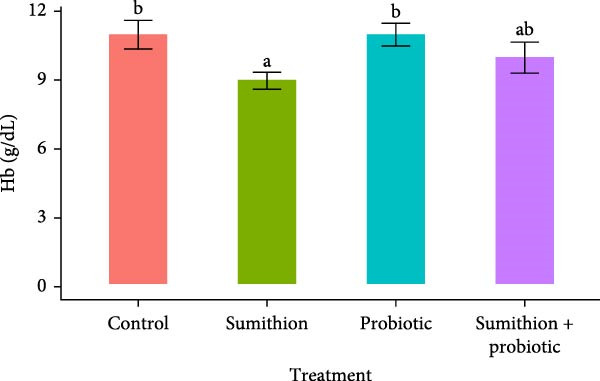
(A)

**Figure:**
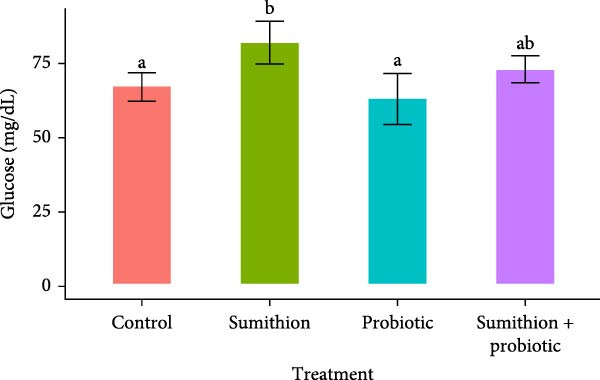
(B)

In contrast, the level of Glu (mg/dL) was statistically higher in T2 (sumithion) in relative to control (T1), which was reduced *p* < 0.05) by probiotic supplementation in T4 (sumithion + probiotics). However, there was no significant difference between T3 (probiotics) and T1 (control) (Figure 1B).

### 3.3. Erythrocytic Cellular and Nuclear Abnormalities

Higher frequencies of cellular abnormalities, such as teardrop, twin, spindle shape, and fusion, in erythrocytes of Nile tilapia (*O. niloticus*) were observed in fishes reared with sumithion (T2) (Figure 2) compared to the control group (T1), depicting the adverse impact of sumithion. The ECAs caused by sumithion were reduced when supplemented with probiotics (T4) (Figure 2). In addition, higher frequencies of spindle‐shaped and fusion cells were evident in all treatments compared to other types of cellular abnormalities. In parallel, ENAs, including nuclear bud, notched nuclei, nuclear bridge, karyopyknosis, were higher in sumithion group (T2) compared to other groups. However, probiotic has lessened the adverse impact of sumithion by reducing the nuclear abnormalities in erythrocytes of Nile tilapia while treated with sumithion + probiotic (T4) (Figure 3). Lower frequencies of nuclear and cellular abnormalities were observed in probiotic treated group (T3) compared to other groups (Figures 2 and 3).

Figure 2(A) Cellular abnormalities (a–d) of erythrocytes of Nile tilapia (*O. niloticus*) reared for 42 days at different treatments: (a) tear‐drop, (b) twin, (c) spindle, and (d) fusion. (B) Alteration in frequencies of erythrocytic cellular abnormalities, (a) tear‐drop, (b) twin, (c) spindle, (d) fusion, and (e) elongated, in Nile tilapia (*O. niloticus*) reared with sumithion, probiotics, and sumithion + probiotics for 42 days.

**Figure:**
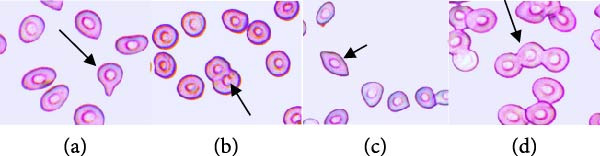
(A)

**Figure:**
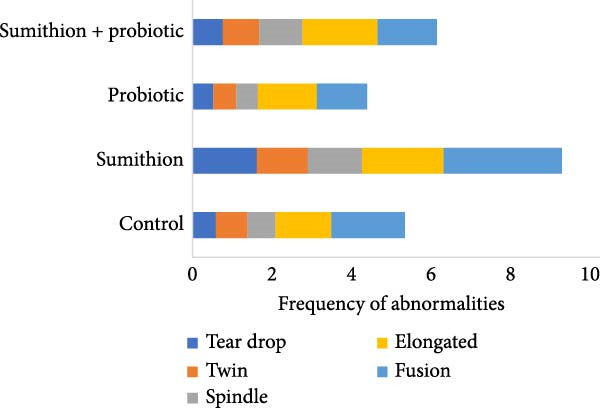
(B)

Figure 3(A) Nuclear abnormalities (a–d) of erythrocytes of Nile tilapia (*O. niloticus*) reared for 42 days at different dietary supplementation: (a) nuclear buds, (b) notched nuclei, (c) nuclear bridge, and (d) karyopyknosis. (B) Alteration in frequencies of erythrocytic nuclear abnormalities: (a) nuclear buds, (b) notched nuclei, (c) nuclear bridge, and (d) karyopyknosis of Nile tilapia (*O. niloticus*) reared with sumithion, probiotics, and sumithion + probiotics for 42 days.

**Figure:**
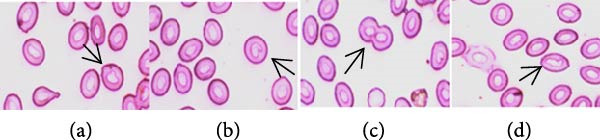
(A)

**Figure:**
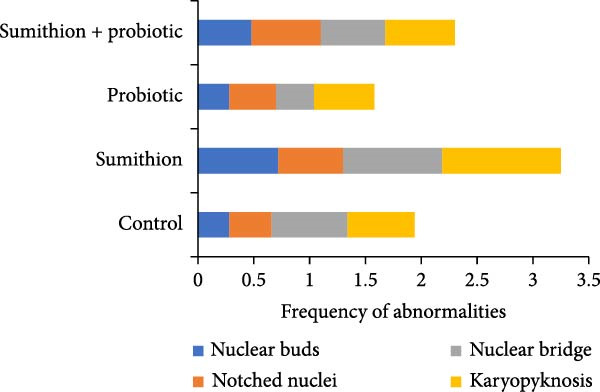
(B)

### 3.4. Changes in Intestinal Morphology

**Figure 4:**
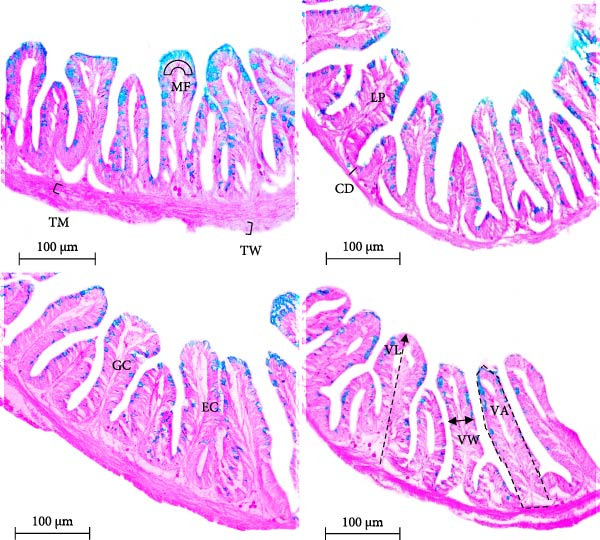
Immune response indicators in gut histology of Nile tilapia (*O. niloticus*) reared with various treatments, for 42 days. CD, crypt depth; EC, enterocyte; GC, goblet cell; LP, lamina propria; MFs, mucosal folds; TM, intestine muscle thickness; TW, wall thickness; VA, villus area; VL, villus length; VW, villus width. Scale bar = 100 µm.

In the present study, histology of the gut samples showed several changes in the intestinal morphological features in *O. niloticus*. Sumithion (T2) exposure significantly (*p* < 0.05) reduced the length and width of villi, thickness of the intestinal wall, and abundance of GCs and enterocytes than control (Figure 4 and Table 3). However, probiotics incorporation (T4) recovered the sumithion‐induced intestinal histological features by altering the length and width of villi, thickness of intestinal wall, and abundance of GCs and enterocytes, making them almost similar to the control group (Figure 4 and Table 3).

**Table 3:** Histological changes in the gut of Nile tilapia (*O. niloticus*) reared with various treatments for 42 days.

Parameters	Treatments			
	Control (T1)	Sumithion (T2)	Probiotics (T3)	Sumithion + probiotics (T4)
Villus length (µm)	68.49 ± 2.70^ab^	58.49 ± 4.63^a^	69.87 ± 5.02^b^	62.11 ± 8.56^ab^
Villus width (µm)	15.80 ± 2.18^b^	7.24 ± 0.94^a^	15.90 ± 1.46^b^	15.08 ± 1.61^b^
Thickness of the wall (µm)	4.95 ± 0.54^a^	3.18 ± 0.33^b^	6.15 ± 0.92^a^	3.61 ± 0.59^b^
Thickness of muscular (µm)	6.11 ± 0.64	5.01 ± 0.28	6.29 ± 0.89	5.80 ± 0.50
Crypt depth (µm)	7.61 ± 0.78	5.73 ± 0.49	7.83 ± 0.82	6.56 ± 1.13
Fattening of mucosal fold (μm)	15.85 ± 2.44	12.52 ± 1.03	16.27 ± 1.49	15.18 ± 1.97
Abundance of goblet cell (GC)	23.30 ± 2.60^b^	13.7 ± 2.22^a^	24.5 ± 3.28^b^	18.00 ± 4.32^ab^
Width of lamina propria (μm)	3.85 ± 0.34	2.77 ± 0.30	3.98 ± 0.95	3.18 ± 0.37
Abundance of enterocytes	11.5 ± 1.84^ab^	9.5 ± 1.51^a^	14 ± 1.76^b^	12.8 ± 1.87^ab^

*Note:* Different lowercase letters in superscript indicate a significant (*p* < 0.05) difference among various treatments.

### 3.5. Expression of Growth‐Related Genes (*gh*, *igf-1*, and *igf-2*) in the Pituitary and Liver

**Figure 5:**
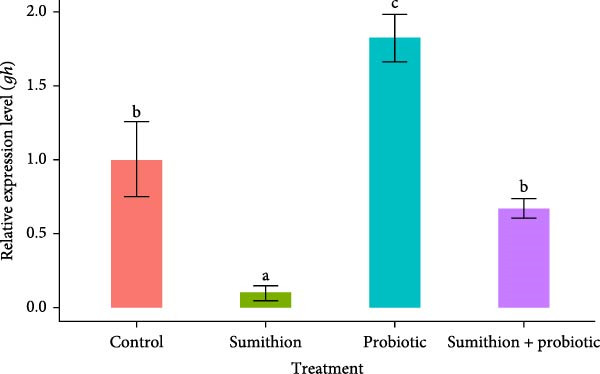
Relative expression of *gh* in the pituitary of Nile tilapia (*O. niloticus*) (*n* = 6) exposed to different treatments (control, sumithion, probiotics, and sumithion + probiotics) for 42 days. Values are presented as mean ± standard deviation (SD). Different lowercase letters indicate a significant (*p* < 0.05) difference among various treatments.

The relative expression level of *gh* in the pituitary was significantly downregulated (*p* < 0.05) in fish reared with sumithion (T2) compared to the control group (T1), indicating an adverse impact of sumithion. In contrast, sumithion exposure combined with probiotics supplementation (T4) had statistically (*p* < 0.05) upregulated the relative expression of *gh*, aligning to the control group (T1), in the pituitary of *O. niloticus* (Figure 5). Moreover, the probiotic‐supplemented (T3) group showed the highest expression level of *gh* in the *O. niloticus* pituitary among all other treatment groups (Figure 5). Similarly, the relative expression of *igf-1* and *igf-2* in the liver was significantly higher (*p* < 0.05) in fish reared with probiotics (T3) among all other groups (Figure 6). On the contrary, the relative expression level of *igf-1* and *igf-2* significantly decreased in fish exposed to sumithion (T2) compared to the control group (T1) (Figure 6). However, fish exposed to sumithion and supplemented with probiotics (T4) had significantly higher expression of *igf-1* and *igf-2* in comparison with sumithion exposure (T2), having no significant difference with the control group (T1) (Figure 6).

Figure 6Relative expression of (A) *igf*‐*1* and (B) *igf*‐*2* in the liver of Nile tilapia (*O. niloticus*) (*n* = 6) exposed to different treatments (control, sumithion, probiotics, and sumithion + probiotics) for 42 days. Values are presented as mean ± standard deviation (SD). Different lowercase letters indicate a significant (*p* < 0.05) difference among various treatments.

**Figure:**
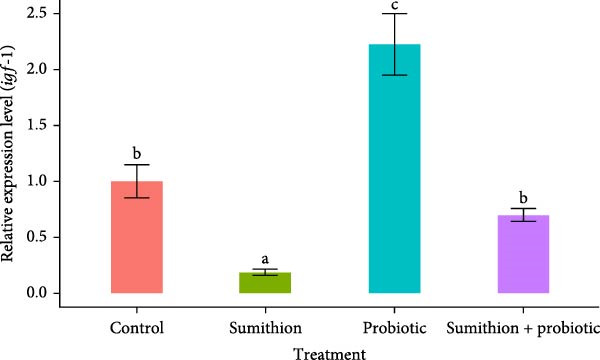
(A)

**Figure:**
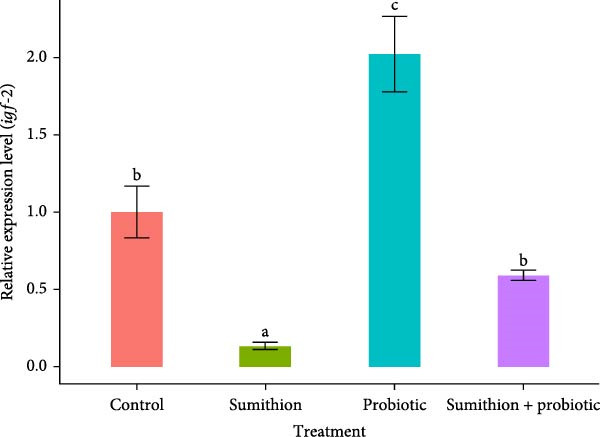
(B)

### 3.6. Expression of Antioxidant‐Related Genes (*SOD* and *CAT*) in the Liver of *O. niloticus*

The relative expression of *SOD* and *CAT* was significantly higher (*p* < 0.05) in fish reared with sumithion (T2) compared to the control group, indicating an increased response to oxidative stress caused by sumithion in the experimental fish (Figure 7). In contrast, the expression level of *SOD* and *CAT* decreased significantly (*p* < 0.05) in the liver of *O. niloticus* while reared with both sumithion and probiotics (T4) than T2 (sumithion), suggesting lower oxidative stress due to probiotics supplementation. However, there was no notable difference observed in the relative mRNA level of *SOD* and *CAT* among the fish in T3 (probiotics), T4 (sumithion + probiotics) and T1 (control) (Figure 7).

Figure 7Relative expression of (A) *SOD* and (B) *CAT* in the liver of Nile tilapia (*O. niloticus*) (*n* = 6) exposed to different treatment (control, sumithion, probiotics, and sumithion + probiotics) for 42 days. Values are presented as mean ± standard deviation (SD). Different lowercase letters indicate a significant (*p* < 0.05) difference among various treatments.

**Figure:**
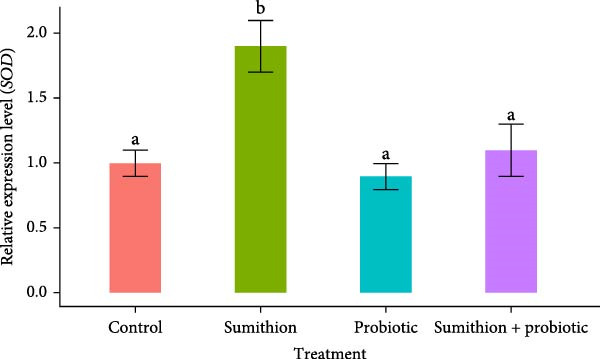
(A)

**Figure:**
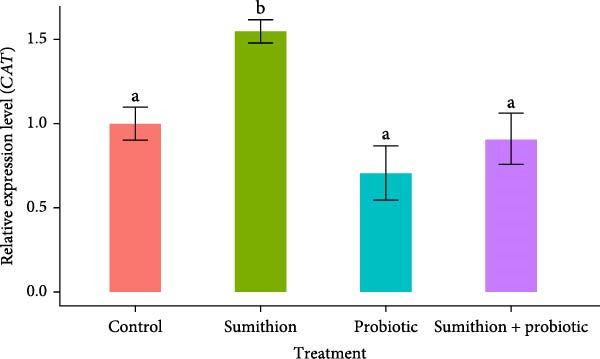
(B)

### 3.7. Expression of Immune‐Related Genes (*TNF-α*, *IL-1β*, and *IFN-γ*) in the Liver of *O. niloticus*

In the present study, the relative expression of *TNF-α*, *IL-1β*, and *IFN-γ* was significantly (*p* < 0.05) downregulated in the sumithion‐exposed group (T2) compared to control (T1) in the liver of *O. niloticus*, depicting an immunosuppressive impact of sumithion (Figure 8). While supplemented with probiotics in sumithion‐exposed group (T4), significantly (*p* < 0.05) higher expression of *TNF-α*, *IFN-γ*, and *IL-1β* was found in the liver of *O. niloticus* in comparison with sumithion‐exposed group (T2) and control (T1), suggesting the counteraction of probiotic to improve immune response in the experimental fish. Moreover, fish in T3 (probiotics) had significantly (*p* < 0.05) higher expression of *TNF-α*, *IL-1β*, and *IFN-γ* than other treatments (Figure 8).

Figure 8Relative expression of (A) *TNF*‐*α*, (B) *IL*‐1*β*, and (C) *IFN*‐*γ* in the liver of Nile tilapia (*O. niloticus*) (*n* = 6) exposed to different treatments (control, sumithion, probiotics, and sumithion + probiotics) for 42 days. Values are presented as mean ± standard deviation (SD). Different lowercase letters indicate a significant (*p* < 0.05) difference among various treatments.

**Figure:**
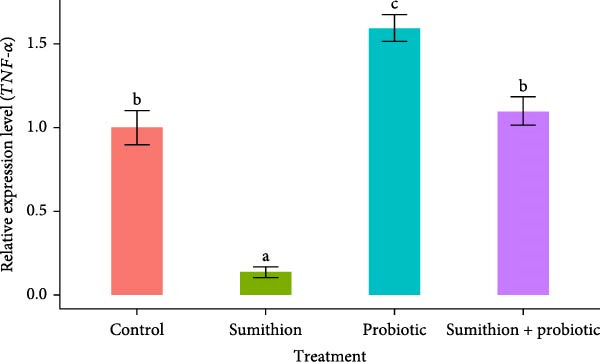
(A)

**Figure:**
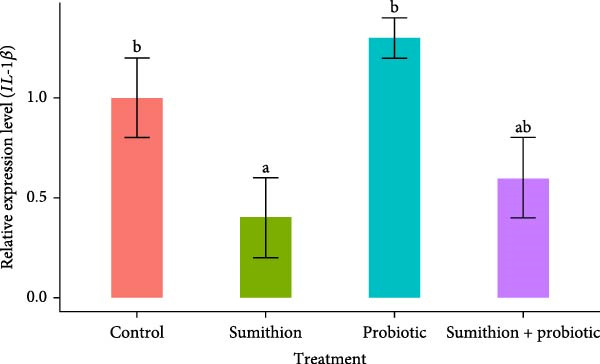
(B)

**Figure:**
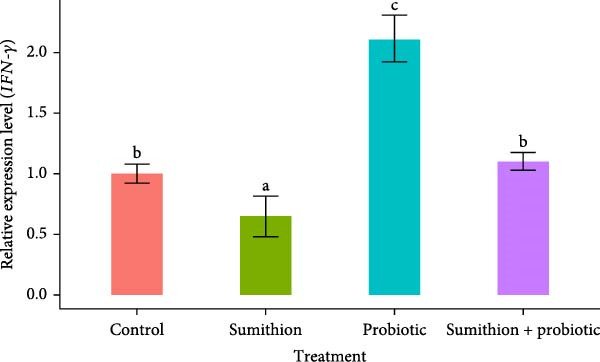
(C)

## 4. Discussion

Pesticides are increasingly recognized as significant environmental stressors that can trigger metabolic, oxidative, and immunological stress and hinder growth in aquatic organisms. In contrast, probiotics supplementation improves the overall well‐being of fish not only in usual conditions but also under stress caused by anomalous temperature, salinity, or heavy metals [45, 46]. In this experiment, Nile tilapia treated with probiotics supplementation under sumithion exposure exhibited significantly better WG and SGR relative to the sumithion‐exposed group. In addition, sumithion‐exposed fish had a significantly higher FCR, which was further reduced while supplemented with probiotics, indicating better feed utilization by probiotics incorporation. Similar results were also found in fish during high temperature exposure [46] and heavy metals [38, 47]. This could be characterized by the increased ability of digestion and absorption of nutrients by influencing digestive enzyme secretion, and increasing the absorption area of the intestine in the presence of probiotics [48, 49], which ultimately improved the growth performance of *O. niloticus* in the present study.

Evaluating hematological and biochemical parameters is recognized as a valuable approach for assessing physiological condition, identifying metabolic disorders, and determining the effects of various stressors in fish and other vertebrates [50, 51]. In the current research, a significant decrease in Hb was noted in sumithion‐exposed fish, which was recovered by probiotics supplementation. Stress tends to hinder the normal mechanisms of the hematopoietic system and reduce Hb level in fish, while probiotics modulate the molecular pathway of hematopoiesis. Thus, sumithion exposure might have resulted in stress and reduced Hb, which is also evident in *Cyprinus carpio* [52], and probiotics enhanced the hematopoietic system functioning by modifying its molecular cascade and upregulated the Hb levels in experimental fish. Oppositely, the Glu content in the blood of *O. niloticus* increased significantly in sumithion‐exposed fish and compensated after supplementing probiotics. Previously, sumithion exposure also increased Glu content in Nile tilapia [53] and *Cyprinus carpio* [52]. Elevated blood Glu levels are considered as a sign of stress [45]. Moreover, fish experiencing stress led to increased stress hormones, including catecholamine, glucocorticoid, and cortisol, stimulating gluconeogenesis for breaking down glycogen to Glu to fulfill high energy requirements and meet the stress‐energy demand [54, 55]. In addition, probiotics play a role in regulating liver glycogen [56], indirectly modulating the Glu level in blood, which in parallel with the present findings of probiotics, serves in lessening higher blood Glu levels induced by sumithion exposure stress. Concomitant results were also reported in *Labeo rohita* and *Pangasianodon hypophthalmus* while exposed to fenvalerate, a synthetic pyrethroid insecticide [45], and salinity stress [39], respectively, then incorporated with multispecies probiotics.

Erythrocytic abnormalities and their associated defects serve as indicators of genotoxicity and oxidative stress induced by toxins across the aquatic ecosystem [5, 57, 58]. As erythrocyte cells contain Hb, involved in maintaining and regulating the hematopoietic system in fish can respond and react to a variety of external stressors, and molecular alterations and modifications of erythrocytes as a result of pesticide exposure [6]. In the current study, fish exposed to sumithion exhibited an increased frequency of a variety of cellular and nuclear abnormalities, indicating the genotoxicity of sumithion. Such alterations might result from an elevation in lipid peroxidation compounds in erythrocytes as a result of toxicity of sumithion [58]. Moreover, small capillaries in the plasma membrane undergo morphological changes due to increased lipid peroxidation, which ultimately make erythrocytes more prone to bursting or losing their shape. In addition, toxic substances can impact the erythrocyte structure, metabolism, and ion permeability of erythrocyte membranes, potentially resulting in the formation of morphologically damaged erythrocytes either directly or indirectly [59]. However, while fish were reared with probiotics during sumithion exposure in the current investigation, the frequency of erythrocytic abnormalities were reduced, suggesting that probiotics might be helpful to reduce oxidative damage and eradicate the pesticide toxicity in fish.

The morphology of the intestine can influence the physiology and metabolism of nutrient absorption which brings about alteration in growth performances outcomes [60]. GCs found in the intestine are responsible for producing and protecting the mucus layer by synthesizing mucins, which are involved in providing both physical and chemical barrier to protect the epithelial layer from enteric and external bacteria, and environmental toxins [61]. Pesticide accumulation in fish can lead to intestinal morphological changes, primarily due to its direct cellular toxicity, concentrated acetylcholine in the tissue, and decreased oxygen availability [62]. In the present study, sumithion exposure reduced the villus length and width, thickness of intestinal wall, and abundance of GCs and enterocytes, which is in agreement with previous research from various studies has highlighted the impacts of different toxicants on the intestines of the fish [63, 64]. However, previous research has also shown that probiotics can improve intestinal conditions by increasing the length and width of villi, intestinal muscles, crypt length, et cetera, in fish [65]. In parallel, these findings show that probiotics application in the sumithion‐exposed group (T4) has improved the intestinal morphological condition by increasing the villus length and width, abundance of GCs and enterocytes close to the control group, displaying the toxicity eradicating potential of probiotics in fish.

The endocrine system is responsible for growth response, including GH and IGF, along with environmental factors serving as important signaling molecules of growth‐related investigation [66]. The somatotropic axis includes GH–IGF axis involved in the primary pathway of growth and tissue proliferation of the vertebrate species [62]. Previous studies showed that various feed additive incorporation affecting growth‐related factors and directly or indirectly influencing GH [67]. In this research, profoundly lower expression of growth‐related genes (*gh*, *igf-1*, and *igf-2*) was observed during sumithion exposure. But probiotics can attenuate the growth factors (GH, IGF‐1, and IGF‐2) regarded as important elements involves in growth and development of fish [68]. Prior research has shown that fish treated with probiotics significantly increased the expression of *gh*, *igf-1*, and *igf-2* in the liver of *Perca flavescens* [69] and *Pagus major* [70]. In this course of investigations, sumithion‐exposed fishes with probiotics supplementation showed a recovering pattern with upregulated expression of growth‐related genes (*gh*, *igf-1*, and *igf-2*), indicating a positive attenuating effect of probiotic on sumithion toxicity by influencing the GH–IGF system, which is also in alliance with the growth performance of *O. niloticus* found in this experiment.

Pesticide exposure leads to oxidative damage in tissues by generating ROS. To combat such oxidative damage, the body activates its antioxidant defense [40]. In the present study, dramatically higher expression of *SOD* and *CAT* in the sumithion‐exposed group, likely to counteract the ROS accumulation in the liver tissue. However, the expression of *SOD* and *CAT* was reduced after probiotics supplementation, approaching values similar to those of control group. Probiotics treatment was found to improve SOD and CAT enzyme activities in *Mugil capito* [71]. Previous study also reported similar result in *Labeo rohita* with upregulation of *SOD* and *CAT* expression in fenvalerate treated group which, however, showed marked recovery by downregulation of *SOD* and *CAT* on the three‐probiotic mixture supplementation [45]. Hence, probiotics might have played role in reducing oxidative damage and ROS production, for which antioxidant‐related gene (*SOD* and *CAT*) expression was downregulated, showcasing positive impact of probiotics on sumithion‐exposed fishes.

Cytokines like *TNF α*, *IL-1β*, and *IFN-γ* are recognized as reliable markers of immune responses in teleost under stress conditions [20]. In the current study, downregulation was recorded for three genes involved in immune response (*TNF α*, *IL-1β*, and *IFN-γ*) in the sumithion‐treated group depicts suppressed activity of inflammation and immunological response in Nile tilapia. Although proinflammatory cytokines are typically upregulated during immune activation, acute exposure to stressor such as pesticides can disrupt immune homeostasis and impair cytokines production [72]. Such stressed condition triggers cortisol mediated immunosuppression and immune cell damage via epigenetic modification, eventually altering gene expression dynamics for immune related cytokines [73]. In contrast, probiotics‐supplemented fish under sumithion exposure gave higher expression of immune‐response genes. Similar findings of elevated immune‐related gene expression were reported in other fish fed with probiotics supplemented diets [74, 75]. Nevertheless, more scientific research exploration is needed to unravel the complex regulatory pathways by which probiotics influence immune‐related gene expression in fish.

Based on this present observation, the adverse impact of sumithion exposure is evident through increased Glu content to meet excess energy demand, upregulated antioxidant‐related genes, reduction in Hb content and enhanced erythrocytic abnormalities in *O. niloticus*. Moreover, downregulated growth and immune response‐related gene expression, and alterations in intestinal morphology during sumithion exposure eventually resulted in reduced growth performance in the present study. However, probiotics can reduce stress by upregulating immunity [27, 39], and species like *Lactobacillus plantarum* and *Bacillus thuringiensis* can degrade organophosphate pesticides [76, 77]. Thus, probiotics, being immunostimulants, reduced sumithion exposure stress by accelerating immune‐response related gene’s expression, reducing ROS production, enhancing growth‐related genes expression and intestinal structure, ultimately resulting in almost normal physiological condition and improved growth in *O. niloticus*, portraying the ability to attenuate the negative impacts of sumithion in fish.

## 5. Conclusions

The aquatic ecosystem is now experiencing greater effects of the indiscriminate use of pesticides. The present findings of our study highlight the detrimental consequences of sumithion on the physiological, hematological, and immunological condition of *O. niloticus*. However, supplementation of probiotics plays a counteracting role against sumithion toxicity in fish by improving immune response, antioxidant capacity, and growth of fish. Thus, probiotics could be an ecofriendly and sustainable solution for overcoming the negative impact of increasing pesticide toxicity in the aquatic ecosystem. This study adds valuable insight into the possible strategies to alleviate the impact of toxicants in aquatic organisms for developing sustainable aquaculture practices. However, future studies should focus on identifying additional effective probiotic strains, determining optimal dosages, and conducting large‐scale field trials to translate laboratory results into practical applications.

## Ethics Statement

The experimental procedure was conducted in accordance with the ethical guidelines approved by the ethical committee of the Bangladesh Agricultural University, Mymensingh (Approval Number: Bau‐FoF/2002/003).

## Consent

The authors have nothing to report.

## Conflicts of Interest

The authors declare no conflicts of interest.

## Author Contributions

Atika Anjum conducted the experiment, methodology, and writing – original draft. Taslima Parvin contributed to data curation and writing – original draft. Shaila Sultana assisted in conducting the experiment, data collection, and formal analysis. Azmaien Naziat assisted in conducting the experiment, data visualization, and validation. Zannatul Ferdous contributed to data collection and curation. Md. Mahiuddin Zahangir contributed to formal analysis, resources, and writing – editing and reviewing. Md Shahjahan contributed to writing – review and editing, supervision, funding acquisition, and conceptualization.

## Funding

The study was conducted with the assistance of funding from BAURES, Bangladesh Agricultural University (Project Number 2024/123/BAU).

## Supporting Information

Additional supporting information can be found online in the Supporting Information section.

## Supporting information


**Supporting Information** Overview of the manuscript in a graphical manner.

## Data Availability

The data that support the findings of this study are available from the corresponding author upon reasonable request.
