# Supplementary material for: Water Added Probiotics Attenuates Sumithion‐Induced Toxicity on Growth, Intestinal Deformities, Erythrocytic Abnormalities, and Immunity in Nile Tilapia (Oreochromis niloticus)
Source: Aquac Nutr. 2026 Jan 16;2026:2689798. doi: 10.1155/anu/2689798 (PMC12811407; doi:10.1155/anu/2689798)
Supplement: Supplementary file 1 — Supporting Information Overview of the manuscript in a graphical manner. [file ANU-2026-2689798-s001.pptx]

## Slide 1
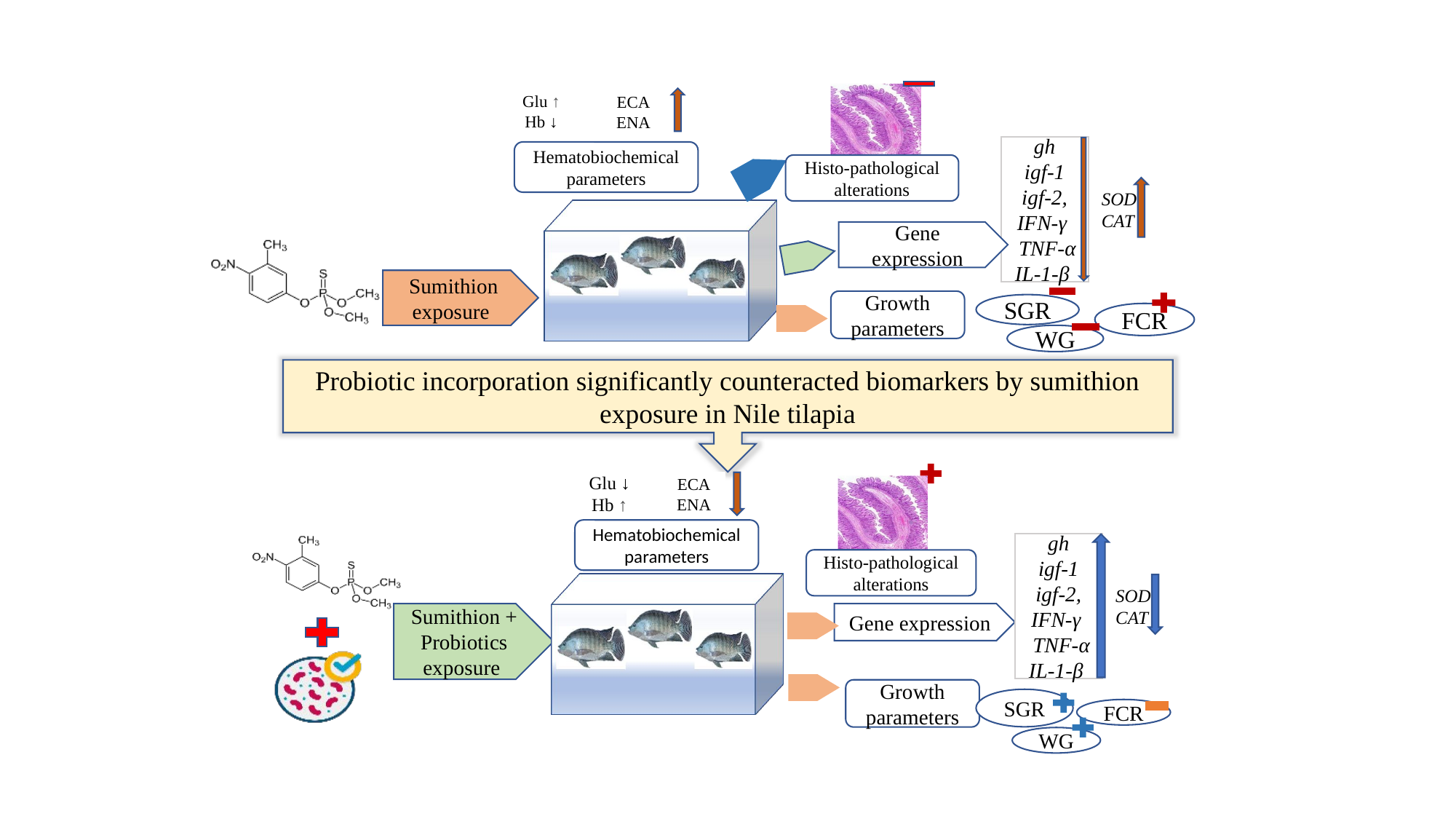

Histo-pathological alterations
ECA
ENA
Glu ↑
Hb ↓
gh
igf-1
 igf-2,
IFN-γ
 TNF-α
IL-1-β
SOD CAT
Hematobiochemical parameters
Sumithion exposure
SGR
FCR
WG
Growth parameters
Probiotic incorporation significantly counteracted biomarkers by sumithion exposure in Nile tilapia
Glu ↓
Hb ↑
ECA
ENA
Histo-pathological alterations
Hematobiochemical parameters
gh
igf-1
 igf-2,
IFN-γ
 TNF-α
IL-1-β
SOD CAT
Sumithion + Probiotics exposure
Gene expression
Growth parameters
SGR
FCR
WG
Gene expression
